# Association of maternal prenatal copper concentration with gestational duration and preterm birth: a multicountry meta-analysis

**DOI:** 10.1016/j.ajcnut.2023.10.011

**Published:** 2023-10-27

**Authors:** Nagendra K. Monangi, Huan Xu, Yue-Mei Fan, Rasheeda Khanam, Waqasuddin Khan, Saikat Deb, Jesmin Pervin, Joan T. Price, Lovejeet Kaur, INTERBIO-21st Study Consortium, Jose Villar, Rose McGready, Fernando C. Barros, Cesar G. Victora, Shama Munim, Aris T. Papageorgh, Roseline Ochieng, Rachel Craik, Hellen C. Barososio, James A. Berkley, Maria Carvalho, Leila Cheikh Ismail, Ann Lambert, Shane A. Norris, Chrystelle OO. Tshivuila-Matela, Francois Nosten, Ricardo Uauy, Zulfiqar A. Bhutta, Stephen Kennedy, Abdullah Al Mahmud, Le Quang Thanh, Angharad Care, Julio A. Landero, Gerald F. Combs, Elizabeth Belling, Joanne Chappell, Jing Chen, Fansheng Kong, Craig Lacher, Salahuddin Ahmed, Nabidul Haque Chowdhury, Sayedur Rahman, Furqan Kabir, Imran Nisar, Aneeta Hotwani, Usma Mehmood, Ambreen Nizar, Javairia Khalid, Usha Dhingra, Arup Dutta, Said Mohamed Ali, Fahad Aftab, Mohammed Hamad Juma, Monjur Rahman, Tahmeed Ahmed, M Munirul Islam, Bellington Vwalika, Patrick Musonda, Ulla Ashorn, Kenneth Maleta, Mikko Hallman, Laura Goodfellow, Juhi K. Gupta, Ana Alfirevic, Susan K. Murphy, Larry Rand, Kelli K. Ryckman, Jeffrey C. Murray, Rajiv Bahl, James A. Litch, Courtney Baruch-Gravett, Shailaja Sopory, Uma Chandra Mouli Natchu, Pavitra V. Kumar, Neha Kumari, Ramachandran Thiruvengadam, Atul Kumar Singh, Pankaj Kumar, GARBH-Ini study team, Zarko Alfirevic, Abdullah H. Baqui, Shinjini Bhatnagar, Jane E. Hirst, Cathrine Hoyo, Fyezah Jehan, Laura Jelliffe-Pawlowski, Anisur Rahman, Daniel E. Roth, Sunil Sazawal, Jeffrey S.A. Stringer, Per Ashorn, Ge Zhang, Louis J. Muglia

**Affiliations:** 41 Nuffield Department of Women’s & Reproductive Health, University of Oxford, Oxford, United Kingdom; 42 Oxford Maternal & Perinatal Health Institute, Green Templeton College, University of Oxford, Oxford, United Kingdom; 43 Shoklo Malaria Research Unit, Mahidol-Oxford Tropical Medicine Research Unit, Faculty of Tropical Medicine, Mahidol University, Mae Sot, Thailand; 44 Centre for Tropical Medicine and Global Health, Nuffield Department of Medicine, Headington, Oxford, United Kingdom; 45 Programa de Pós-Graduação em Saúde e Comportamento, Universidade Católica de Pelotas, Pelotas, Brazil; 46 Programa de Pós-Graduação em Epidemiologia, Universidade Federal de Pelotas, Pelotas, Brazil; 47 Department of Obstetrics and Gynaecology, Division of Women and Child Health, Aga Khan University, Karachi, Pakistan; 48 Faculty of Health Sciences, Aga Khan University, Nairobi, Kenya; 49 KEMRI-Centre for Global Health Research, Liverpool School of Tropical Medicine; 50 KEMRI/Wellcome Trust Research Programme, Kenya; 51 Clinical Nutrition and Dietetics Department, University of Sharjah, Sharjah, United Arab Emirates; 52 SAMRC Developmental Pathways For Health Research Unit, Department of Paediatrics & Child Health, University of the Witwatersrand, Johannesburg, South Africa; 53 Department of Nutrition and Public Health Interventions Research, London School of Hygiene and Tropical Medicine; 1 Division of Neonatology, Cincinnati Children's Hospital Medical Center, Cincinnati, OH, United States; 2 Center for Prevention of Preterm Birth, Perinatal Institute, Cincinnati Children's Hospital Medical Center and March of Dimes Prematurity Research Center Ohio Collaborative, Cincinnati, Ohio, United States; 3 Department of Pediatrics, University of Cincinnati College of Medicine, Cincinnati, OH, United States; 4 Division of Human Genetics, Cincinnati Children's Hospital Medical Center, Cincinnati, OH, United States; 5 Center for Child, Adolescent and Maternal Health Research, Faculty of Medicine and Health Technology, Tampere University, Tampere, Finland; 6 International Center for Maternal and Newborn Health, Department of International Health, Johns Hopkins Bloomberg School of Public Health, Baltimore, Maryland, United States; 7 Biorepository and Omics Research Group, Department of Pediatrics and Child Health, Faculty of Health Sciences, Medical College, Aga Khan University, Karachi, Sindh, Pakistan; 8 Research Division, Public Health Laboratory, Center for Public Health Kinetics, Chake Chake, Tanzania; 9 Maternal and Child Health Division, International Centre for Diarrheal Disease Research, Bangladesh, Dhaka District, Bangladesh; 10 Obstetrics and Gynecology, University of North Carolina at Chapel Hill, Chapel Hill, North Carolina, United States; 11 Child and Maternal Health Program, Translational Health Science and Technology Institute (THSTI), Faridabad, India; 12 Nutrition and Clinical Services Division, International Centre for Diarrheal Disease Research, Bangladesh, Dhaka, Bangladesh; 13 Tu Du Hospital, Ho Chi Ming City, Vietnam; 14 Department of Women's and Children's Health, The University of Liverpool, Liverpool, United Kingdom; 15 Department of Chemistry, University of Cincinnati, Cincinnati, OH, United States; 16 Jean Mayer USDA Human Nutrition Research Center on Aging, Tufts University, Boston, MA, United States; 17 Division of Biomedical Informatics, Cincinnati Children’s Hospital Medical Center, Cincinnati, Ohio; 18 USDA-ARS, Grand Forks Human Nutrition Research Center, Grand Forks, ND, United States; 19 Projahnmo Research Foundation, Dhaka, Bangladesh; 20 Center for Public Health Kinetics, New Delhi, India; 21 School of Medicine, University of Zambia, Lusaka, Zambia; 22 School of Public Health, University of Zambia, Lusaka, Zambia; 23 School of Public Health and Family Medicine, University of Malawi College of Medicine, Blantyre, Malawi; 24 Medical Research Centre Oulu, PEDEGO Research Unit, University of Oulu, Oulu, Pohjois-Pohjanmaa, Finland; 25 Department of Obstetrics and Gynecology, Duke University Medical Center, Durham, NC, United States; 26 Department of Obstetrics, Gynecology and Reproductive Sciences, University of California San Francisco School of Medicine, San Francisco, CA, United States; 27 Department of Epidemiology, University of Iowa College of Public Health, Iowa City, IA, United States; 28 Department of Pediatrics, University of Iowa, Iowa City, IA, United States; 29 Department of Maternal, Newborn, Child and Adolescent Health, World Health Organization, Geneva, Switzerland; 30 Global Alliance to Prevent Prematurity and Stillbirth, Lynnwood, WA, United States; 31 St. John's Research Institute (SJRI), Bangladore, India; 32 Geochronology Group, Inter University Accelerator Centre (IUAC), Delhi, India; 33 Nuffield Department of Women’s & Reproductive Health, University of Oxford, Oxford, United Kingdom; 34 Department of Biological Sciences and Center for Human Health and the Environment, North Carolina State University, Raleigh, North Carolina, United States; 35 Department of Pediatrics and Child Health, Aga Khan University, Karachi, Pakistan; 36 Department of Epidemiology and Biostatistics, University of California San Francisco School of Medicine, San Francisco, CA, United States; 37 Centre for Global Child Health, Hospital for Sick Children, University of Toronto, Toronto, Canada; 38 Department of Pediatrics, University of Toronto, Toronto, Canada; 39 Department of Pediatrics, Tampere University Hospital, Tampere, Finland; 40 Burroughs Wellcome Fund, Research Triangle Park, NC, United States

**Keywords:** nutrition, pregnancy, low- and middle-income countries, copper, preterm birth, gestational duration, inflammation, acute-phase reactants

## Abstract

**Background:**

Copper (Cu), an essential trace mineral regulating multiple actions of inflammation and oxidative stress, has been implicated in risk for preterm birth (PTB).

**Objectives:**

This study aimed to determine the association of maternal Cu concentration during pregnancy with PTB risk and gestational duration in a large multicohort study including diverse populations.

**Methods:**

Maternal plasma or serum samples of 10,449 singleton live births were obtained from 18 geographically diverse study cohorts. Maternal Cu concentrations were determined using inductively coupled plasma mass spectrometry. The associations of maternal Cu with PTB and gestational duration were analyzed using logistic and linear regressions for each cohort. The estimates were then combined using meta-analysis. Associations between maternal Cu and acute-phase reactants (APRs) and infection status were analyzed in 1239 samples from the Malawi cohort.

**Results:**

The maternal prenatal Cu concentration in our study samples followed normal distribution with mean of 1.92 μg/mL and standard deviation of 0.43 μg/mL, and Cu concentrations increased with gestational age up to 20 wk. The random-effect meta-analysis across 18 cohorts revealed that 1 μg/mL increase in maternal Cu concentration was associated with higher risk of PTB with odds ratio of 1.30 (95% confidence interval [CI]: 1.08, 1.57) and shorter gestational duration of 1.64 d (95% CI: 0.56, 2.73). In the Malawi cohort, higher maternal Cu concentration, concentrations of multiple APRs, and infections (malaria and HIV) were correlated and associated with greater risk of PTB and shorter gestational duration.

**Conclusions:**

Our study supports robust negative association between maternal Cu and gestational duration and positive association with risk for PTB. Cu concentration was strongly correlated with APRs and infection status suggesting its potential role in inflammation, a pathway implicated in the mechanisms of PTB. Therefore, maternal Cu could be used as potential marker of integrated inflammatory pathways during pregnancy and risk for PTB.

## Introduction

Preterm birth (PTB), defined as birth before 37 completed wk (259 d) of gestation, is the leading cause of perinatal morbidity and mortality worldwide [1]. Globally, it is estimated that approximately 15 million babies are born preterm every year, with an average PTB rate of approximately 11% [2], ranging from 5% to 18%, with higher rates occurring in sub-Saharan African and South Asian low- and middle-income countries. Despite the global burden, the underlying drivers of PTB are uncertain. In particular, little is known about the role of maternal essential trace metals in contributing to or predicting PTB.

Copper (Cu) is an essential trace element regulating several critical biological processes through incorporation into Cu-dependent proteins. These cuproproteins serve critical cellular homeostatic functions in maintaining redox status and antioxidant defenses and modulating inflammatory processes [[3], [4], [5], [6], [7]]. Ceruloplasmin is the major Cu-carrying protein in the blood, carrying approximately 75% to 95% of circulating Cu [8]. The main function of ceruloplasmin is to oxidize ferrous iron (Fe^2+^) to the less damaging ferric iron (Fe^3+^), which enables the iron to be bound by transferrin, the major iron-transport protein. Cu in the form of ceruloplasmin possesses antioxidant activity by preventing free radical damage [8]. Cu also has multiple actions implicated in the modulation of inflammation including the rise in ceruloplasmin [9,10] whose expression levels increase during infection, stress, and inflammation. Ceruloplasmin is an acute-phase reactant (APR) protein, the expression of which increases with systemic inflammation similar to C-reactive protein (CRP) and α1-acid glycoprotein (AGP) [11]. Animal studies have demonstrated the role of Cu in modulation of inflammation and lipid peroxidation [10,[12], [13], [14]]. Spontaneous PTB is thought to be prompted by a cascade of inflammatory events, leading to cytokine upregulation and subsequent induction of uterine activity by promoting the expression and release of uterotonic factors [15]. Maternal Cu concentration during pregnancy increases through early pregnancy and reportedly plateaus by mid-second trimester [16]. Recently, some studies have suggested higher maternal or cord blood Cu levels were associated with increased risk of PTB [17,18] while some others suggested an increased risk of PTB with Cu deficiency [19,20].

In this study, we aimed to examine the association of maternal Cu concentration during early- and mid-pregnancy with PTB risk and gestational duration in a large number of samples collected from geographically diverse study cohorts with different social and ancestral backgrounds and varying degrees of environmental exposures. We leveraged the APRs and infection (malaria and HIV) data collected from pregnant women at the same gestational age as Cu concentration from the Malawi cohort to investigate the correlations between maternal Cu concentration and inflammation and infection.

## Methods

### Study design and participants

**FIGURE 1:**
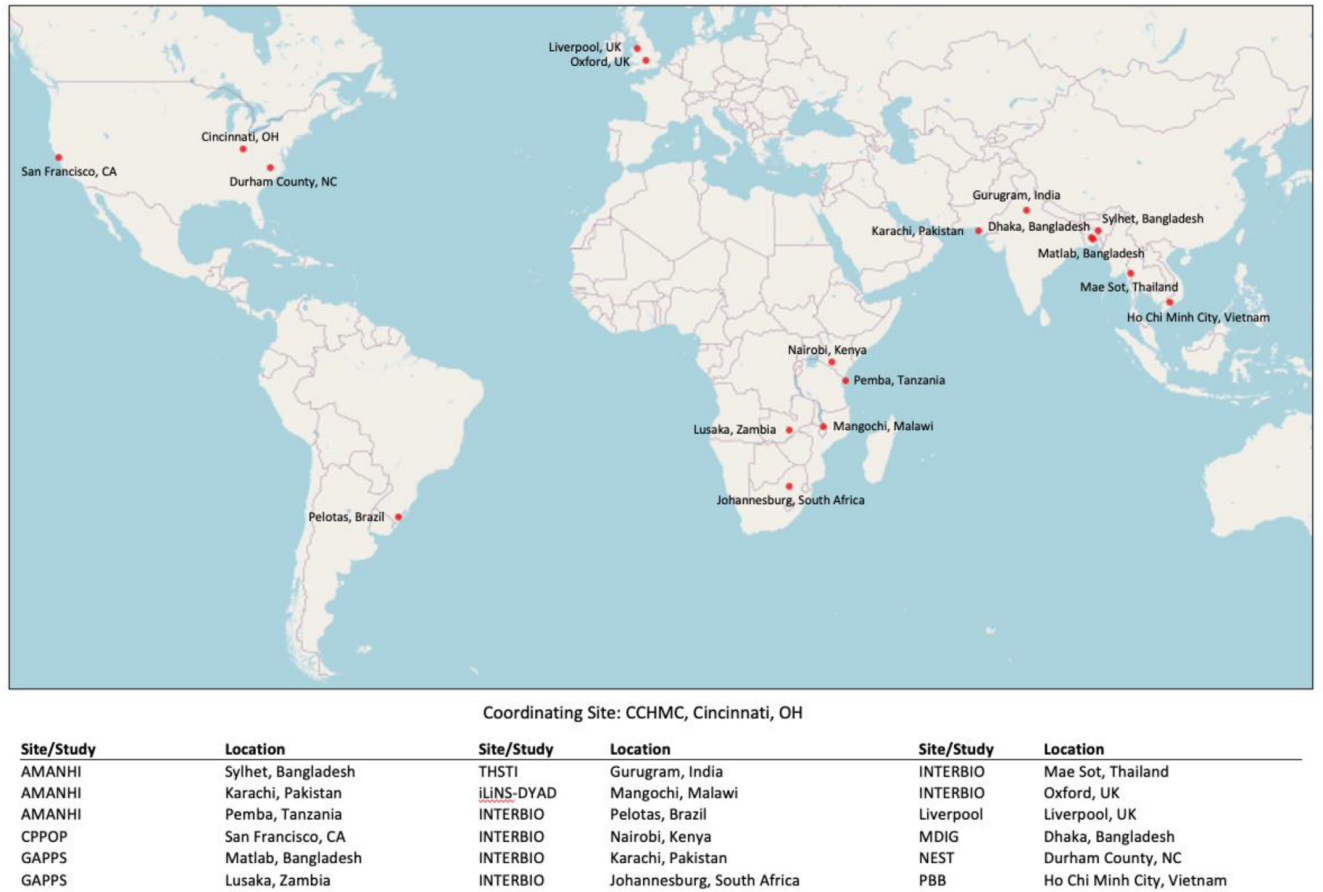
Geographic location of study sites.

The International Consortium on Selenium, Genetics and Preterm Birth is a Bill & Melinda Gates Foundation funded project to study the possible associations between maternal prenatal trace metals and risk of PTB using data and samples from established diverse birth cohorts worldwide. The consortium comprises 18 international pregnancy cohorts across a wide geographic distribution (Figure 1) with Cincinnati Children’s Hospital Medical Center (CCHMC) serving as the coordinating hub [21]. Our study protocol was approved by the Institute Review Board of the CCHMC and by the corresponding Ethics Committees of each participating institution. All study participants provided informed consent as required by each study protocol. Among the participating sites, the Malawi (iLiNS-DYAD) [22] and Bangladesh (MDIG) [23] cohorts were intervention trials and United States, CA (CPPOP) was a case–control study. All the other cohorts were designed to enroll eligible pregnant women in the community or at hospitals. The description and study characteristics of these cohorts are provided in Supplemental Text 1 and Supplemental Table 1.

### Samples and sampling data

Demographic, prenatal, delivery and fetal/newborn data (Supplemental Table 2) as collected by the individual sites according to their local protocols were shared with the coordinating hub (CCHMC). The data collected from Bangladesh (GAPPS), Bangladesh (MDIG) [23], Vietnam (PBB), United States (NEST; CPPOP) [24,25], and all AMANHI cohorts [26] were case–control (preterm/term) samples. The data collected from other sites, including Malawi (iLiNS-DYAD) [22], Zambia (GAPPS), India (THSTI) [27], and the 6 INTERBIO-21st sites [28] were population- or hospital-based samples. Gestational age dating was assigned at the site level by ultrasound, last menstrual period (LMP), or both (Supplemental Table 1). Preterm cases were defined as birth prior to 37 wk of gestation and term controls as birth at 37 wk or later. We excluded stillbirths and multigestational pregnancies.

### Cu measurement

Cu concentrations were measured in maternal plasma or serum [29] stored at −70°C or −80°C freezers before and after use at the CCHMC Biobank (Supplemental Table 1). To mitigate the potential batch effect, samples from each site were randomized prior to analysis in batches. Inductively coupled plasma mass spectrometry (ICP-MS) measurements of Cu concentrations were performed using Agilent 7700 ICP-MS (Agilent Technologies) at the laboratory of Clinical Chemistry and Biochemistry, the University of Cincinnati as described in detail in the protocol (Supplemental Text 2). The samples from India (THSTI) were analyzed at the Inter University Accelerator Center at National Geochronology Facility (Delhi, India) using the same method and protocol as CCHMC. The samples from Bangladesh (MDIG) were analyzed at the Centers for Disease Control and Prevention (Atlanta, GA).

### Acute-phase reactants in Malawi cohort

We obtained plasma concentrations of 3 APRs (CRP, AGP, and albumin [ALB]) measured in Malawi (iLiNS-DYAD) cohort samples collected at the same time for Cu measurement. Plasma concentrations of CRP and AGP were measured by immunoassay using a COBAS Integra Analyzer (Roche Diagnostics). All samples were analyzed singularly, except for 5%, which were randomly selected to be analyzed in duplicate. None of the samples analyzed in duplicate had a coefficient of variation greater than 5%. HIV infection at study enrollment was tested with a whole-blood antibody rapid test (Alere Determine HIV-1/2; Alere Medical). Malaria during pregnancy was diagnosed on-site from finger-prick blood samples using the rapid diagnostic test Clearview Malaria Combo (British Biocell International).

### Statistical analysis

Phenotypic data from participating study sites were harmonized by applying a uniform data structure and consistent coding rules for key variables (e.g, gestational duration, maternal age, maternal height, and fetal sex). The distributions of gestational duration and Cu concentrations at each site were visually inspected using histograms and violin plots. Outliers for gestational duration and Cu concentrations were detected based on fitting with appropriate probability distributions and removed from further association analysis. Specifically, we first fitted several candidate distributions (normal, lognormal, Weibull, Cauchy) to the data and selected the best fitting distribution based on goodness of fit. We then determined observations to be outliers if their probability under the fitted distribution was extremely low (*P* < 0.01/*n*, where *n* is the sample size).

To determine the covariates to be included in the association analysis, we first examined the correlation of PTB and gestational duration with other covariates as well as that between Cu concentration and other covariates at each site using Pearson correlation. The DerSimonian-Laird (DSL) random-effect meta-analysis was used to combine the correlation coefficients across the study sites. Variables significantly correlated (*P* < 0.05) with either PTB or gestational duration or Cu concentration were included as covariates. For each site, we estimated the association between maternal Cu concentration and PTB (and gestational duration as a continuous variable) using logistic (for PTB) or linear (for gestational duration) regression analysis with selected covariates. Random-effect meta-analysis was used to combine the results from different cohorts, and between-study heterogeneity was checked using Cochran’s Q test. Some of the cohorts used case/control samples (Supplemental Table 1 and Supplemental Table 3) with different case/control ratios. Because regression analysis of gestational duration as a continuous variable in nonrandom samples could potentially introduce bias in effect-size estimation, we conducted regression analysis weighted by the inverse of sampling probability (IPW) based on their case/control status (Supplemental Table 3).

All analyses were performed with Microsoft R Open 4.0.2. The cross-site meta-analysis of associations and correlations were conducted using metafor and metocor packages.

## Results

### Gestational duration, PTB, and their correlations with other covariates

**FIGURE 2:**
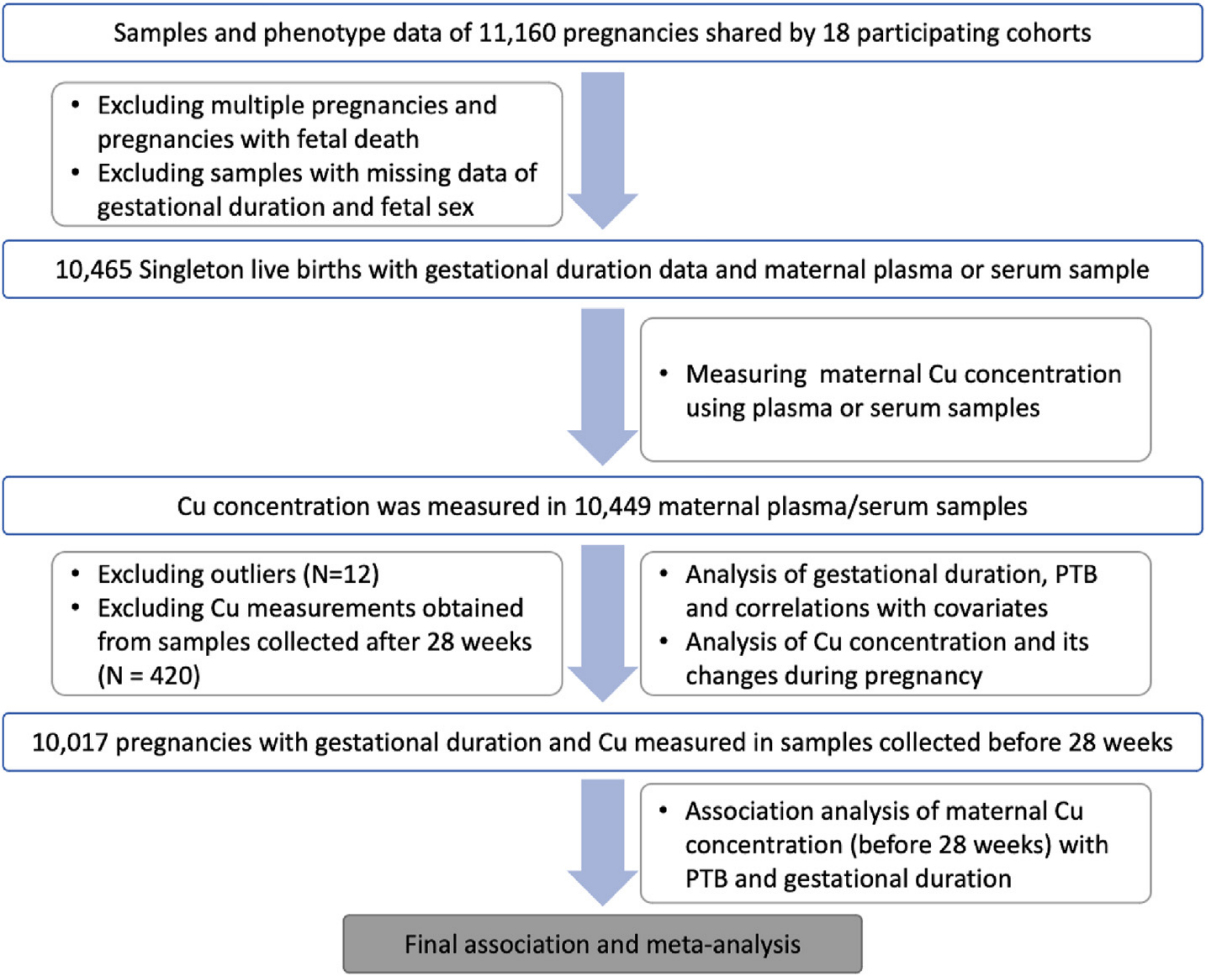
Flow chart of the study illustrating the total number of subjects and inclusion/exclusion criteria. The boxes on the left side list the exclusion criteria applied at different stages, and the boxes on the right side describe the experiment and data analyses performed at different stages.

Pregnancy phenotype and birth outcomes of 11,160 pregnancies were obtained from 18 study sites (Table 1, Supplemental Table 2). Among these, 10,449 singleton live births had a gestational age estimated in days (gday) and maternal plasma or serum Cu concentrations measured (Figure 2). The characteristics of these mothers (e.g., age, height and gestational duration) are summarized by site (Table 1). After removing 3 outliers, the gestational duration followed a Weibull distribution with a mean of 268 d and a median of 273 d ranging from 147 to 312 d (distribution parameters: shape: 21.1, scale: 276.0) (Supplemental Figure 1). The distributions of gestational days in term (gday ≥ 259 d) and preterm (gday < 259 d) births from each site are shown in Supplemental Figure 2.

**TABLE 1:** Demographic characteristics of study subjects

Site	Sample size	Preterm		Sex		Maternal age		Maternal height		GA at delivery		GA at sampling		Birth weight	
		Term	Preterm	Male	Female	Mean	SD	Mean	SD	Mean	SD	Mean	SD	Mean	SD
Bangladesh (AMANHI)	506	253 (50.0%)	253 (50.0%)	239 (47.2%)	267 (52.8%)	23.6	4.5	149.2	5.6	260.7	20	95.8	23.1	2516.1	495.2
Bangladesh (GAPPS)	258	172 (66.7%)	86 (33.3%)	132 (51.2%)	126 (48.8%)	23.9	5.9	151.8	5.5	267.8	18.7	158.8	4.7	2722.7	581.1
Bangladesh (MDIG)	205	135 (65.9%)	70 (34.1%)	105 (51.2%)	100 (48.8%)	23.4	4.5	151.4	5.6	265.7	14.3	143.9	13.5	2664.6	378.4
Brazil (INTERBIO)	389	344 (88.4%)	45 (11.6%)	212 (54.5%)	177 (45.5%)	28.5	5.4	162.5	6.4	270.4	10.8	132.2	53.9	3147.1	464.3
India (THSTI)	506	435 (86.0%)	71 (14.0%)	281 (55.5%)	225 (44.5%)	23.4	3.8	153.2	6	271.1	14.4	91.6	25.1	2744.8	492.2
Kenya (INTERBIO)	553	528 (95.5%)	25 (4.5%)	293 (53.0%)	260 (47.0%)	30.4	4.1	161.8	5.8	278.3	11.1	112.5	40.6	3267	463.8
Malawi (iLiNS-DYAD)	1212	1120 (92.4%)	92 (7.6%)	587 (48.4%)	625 (51.6%)	25.2	6.2	156.1	5.7	276	14.3	117.7	14.9	2976.6	449.5
Pakistan (AMANHI)	348	233 (67.0%)	115 (33.0%)	189 (54.3%)	159 (45.7%)	26.3	5.1	154.8	6.1	265.5	16.5	95.1	24.6	2684.4	500.1
Pakistan (INTERBIO)	516	413 (80.0%)	103 (20.0%)	251 (48.6%)	265 (51.4%)	30.1	4.6	158	5.9	264.9	13.5	103.5	35.6	2876.5	480.7
South Africa (INTERBIO)	352	299 (84.9%)	53 (15.1%)	181 (51.4%)	171 (48.6%)	30.2	5.8	159	6.9	269.4	17.5	88.2	19.6	2940.3	588.8
Tanzania (AMANHI)	351	234 (66.7%)	117 (33.3%)	174 (49.6%)	177 (50.4%)	27.9	6.6	155.2	5.9	267.5	19.6	99.1	23.1	3111.9	592.5
Thailand (INTERBIO)	514	485 (94.4%)	29 (5.6%)	266 (51.8%)	248 (48.2%)	26.2	6.1	151.8	5.1	275.6	11.5	114.4	37.1	2965.8	457.3
United Kingdom (INTERBIO)	648	594 (91.7%)	54 (8.3%)	342 (52.8%)	306 (47.2%)	31.1	4.8	165.3	6.5	275.9	14.6	89.9	20.3	3301.1	586
United Kingdom (Liverpool)	525	424 (80.8%)	101 (19.2%)	271 (51.6%)	254 (48.4%)	30.6	4.9	164.8	6.3	267	21.7	140.8	9.5	3141.2	730.3
United States, California (CPPOP)	966	484 (50.1%)	482 (49.9%)	505 (52.3%)	461 (47.7%)	30	6.1	161.6	7.3	249.8	29.9	115.7	7.7	2763.1	923
United States, North Carolina (NEST)	657	438 (66.7%)	219 (33.3%)	363 (55.3%)	294 (44.7%)	28.3	6.1	162.8	7.7	263.1	22.5	161.1	90.5	2999.6	750.4
Vietnam (PBB)	970	651 (67.1%)	319 (32.9%)	495 (51.0%)	475 (49.0%)	29.1	4.6	156	4.8	264.4	18.9	149	6.2	2959.9	613.5
Zambia (GAPPS)	973	853 (87.7%)	120 (12.3%)	478 (49.1%)	495 (50.9%)	27.7	5.8	160.4	6.5	271.6	18.2	137.3	27.2	3008.7	591.7
TOTAL	10449	8095 (77.5%)	2354 (22.5%)	5364 (51.3%)	5085 (48.7%)	27.8	5.9	158.2	7.6	267.9	19.9	120.6	39.8	2956.5	632.9

GA, gestational age.

We examined the correlation of PTB and gestational duration with other covariates (maternal age, height, fetal sex, and gestational age at sampling) at each participant site (Supplemental Figure 3). Meta-analysis using the DSL method showed that PTB risk was significantly correlated with maternal height and fetal sex. Similarly, gestational duration was also significantly correlated with maternal height (shorter mothers had shorter gestational duration) and fetal sex (males had shorter gestational duration).

### Maternal prenatal Cu concentration and its correlations with other covariates

Cu concentrations were successfully measured in 10,449 mothers. After excluding 12 outliers (3 outliers for gestational duration and 9 outliers for Cu concentration), the Cu concentrations followed a normal distribution (Supplemental Figure 4) with a mean of 1.92 μg/mL and standard deviation of 0.43 μg/mL. The Cu concentrations varied across different sites (Supplemental Figure 5, Supplemental Figure 6) and across different experimental batches for each site (Supplemental Figure 7). The highest average Cu was observed in the Bangladesh (MDIG) cohort with a mean level of 2.2 μg/mL, and the lowest Cu was in the Bangladesh (AMANHI) cohort with a mean concentration of 1.4 μg/mL (Table 2). The Cu concentrations increased with gestational age (see below); however, even after adjustment for gestational age at sampling, the Cu concentrations still showed between-site differences (Supplemental Figure 5B).

**TABLE 2:** Summary statistics of maternal copper (Cu) concentration at different study sites

Site	*n*	Mean	SD	Median	Min.	Max.
Bangladesh (AMANHI)	506	1.36	0.34	1.34	0.62	2.34
Bangladesh (GAPPS)	258	1.85	0.32	1.82	0.97	3.22
Bangladesh (MDIG)	205	2.21	0.4	2.18	1.06	3.48
Brazil (INTERBIO)	389	1.81	0.34	1.8	0.84	3.45
India (THSTI)	504	1.7	0.48	1.67	0.16	3.74
Kenya (INTERBIO)	553	1.94	0.37	1.92	0.99	3.63
Malawi (iLiNS-DYAD)	1211	2.01	0.36	1.99	0.93	3.42
Pakistan (AMANHI)	346	2.01	0.5	1.95	0.96	3.72
Pakistan (INTERBIO)	516	1.95	0.45	1.93	0.84	3.83
South Africa (INTERBIO)	352	1.99	0.41	1.96	0.82	3.44
Tanzania (AMANHI)	350	1.88	0.4	1.87	0.9	3.16
Thailand (INTERBIO)	514	1.7	0.36	1.7	0.63	3.55
United Kingdom (INTERBIO)	644	1.74	0.38	1.71	0.69	3.17
United Kingdom (Liverpool)	525	2.1	0.41	2.06	0.23	3.57
United States, California (CPPOP)	966	2.08	0.37	2.07	0.14	3.57
United States, North Carolina (NEST)	657	1.98	0.49	1.96	0.23	3.65
Vietnam (PBB)	969	1.98	0.39	1.94	1.04	3.8
Zambia (GAPPS)	972	2.05	0.35	2.02	1.11	3.75
TOTAL	10,437^1^	1.92	0.43	1.91	0.14	3.83

1 The total number of samples with maternal Cu concentrations measured was 10,449. A total of 12 outliers were excluded: 3 for gestational duration and 9 for Cu concentration.

We examined the correlation of maternal Cu concentration with other covariates in each site. When combined across sites, the Cu concentration across sites was significantly positively correlated with maternal age (ρ = 0.05, *P* = 0.0017) and negatively correlated with maternal height (ρ = −0.05, *P* = 0.0001) and was higher in mothers with female babies (*P* = 0.01). Cu concentration was also positively correlated with selenium concentration measured from the same samples (ρ = 0.14, *P* = 0.0014) (selenium data was not available in THSTI samples). In addition, there was a strong positive correlation between Cu concentration and gestational age at the time of sample collection (ρ = 0.28, *P* = 3.3e-9) (Supplemental Figure 8).

### Change of maternal prenatal Cu concentration during pregnancy

**FIGURE 3:**
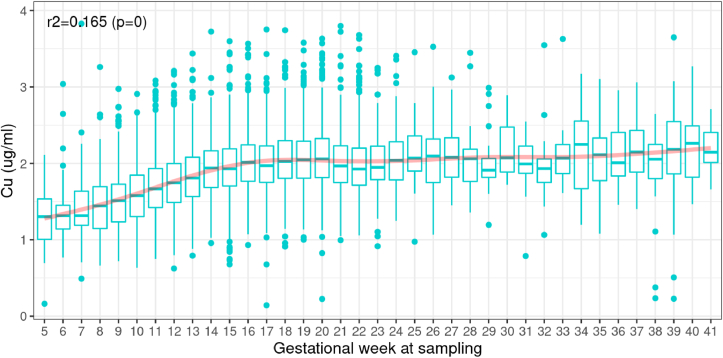
Copper concentration at each gestational week of sampling from 5 to 41 wk (*N* = 10,432).

To inspect the change of maternal Cu concentration visually across different gestational ages, we generated boxplots of Cu concentration at each week of gestational age of sampling for the 10,432 samples collected between 5 wk and 41 wk (Figure 3). The mean maternal Cu concentration increased substantially during early gestation, from a mean at 1.3 μg/mL to 2.0 μg/mL between week 5 to week 16 and plateaued and gradually reached 2.2 μg/mL near delivery, with some fluctuations. Given the nonlinear relationship between maternal Cu and gestational age at sampling, the first 2 polynomials of gestational age at sampling were included in later regression analysis.

The gestational age at sample collection varied substantially from site to site, and at some sites, there were some samples collected after the second trimester (≥28 wk gestation) (Supplemental Figure 9). To minimize the bias introduced by these samples (e.g., exclusion of extremely PTB and the nonlinear increase of maternal Cu concentration), we excluded 420 samples that were collected at 28 wk gestation or later (including 4 samples without a known date of sample collection) from the final association analysis.

### Association of maternal Cu concentration with PTB and gestational duration

**FIGURE 4:**
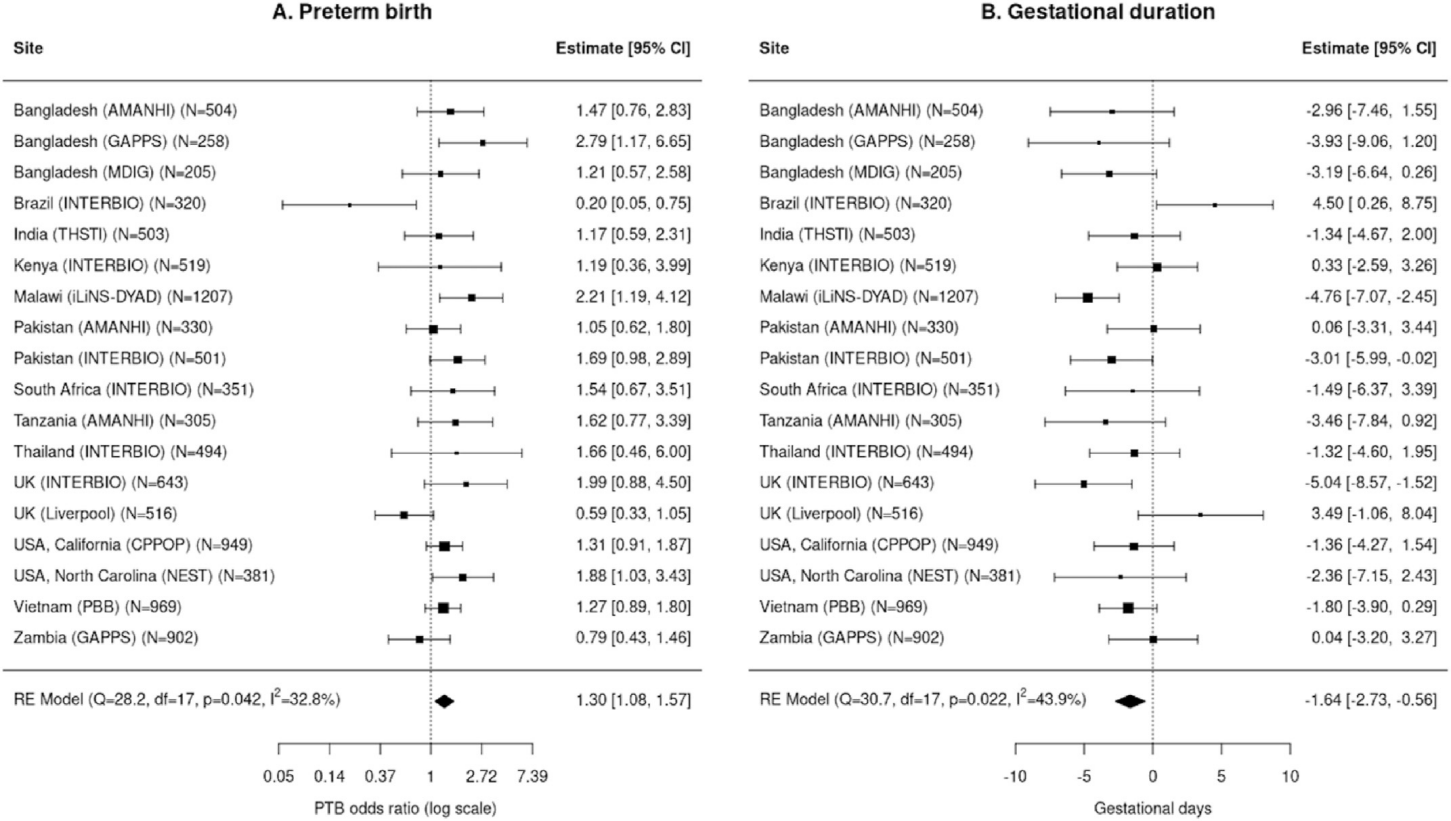
Meta-analysis of the association of maternal copper concentration with PTB (A) and gestational duration (B). This analysis was performed in 10,017 pregnancies with samples collected before 28 wk.

We examined the association of maternal Cu concentration before the third trimester with gestational age at sample collection <28 wk with PTB and gestational duration in each individual site and then combined the results using meta-analysis (Figure 4). In total, the associations were tested in 10,017 pregnancies (Figure 2). The covariate factors that were found to be significantly associated (*P* < 0.05) with either gestational duration or Cu concentration were incorporated as covariates. These included maternal age, maternal height, fetal sex, experimental batch, and the first 2 polynomials of gestational days at sample collection. Given the enrichment of preterm cases in the case–control studies that could potentially introduce bias, we conducted the IPW analysis in the 8 case/control data sets in the regression analysis of gestational duration to correct for the sampling bias (Figure 4B, Supplemental Table 3).

In the combined meta-analysis and in several of the individual cohorts, higher maternal Cu concentration was associated with higher risk of PTB and shorter gestational duration, except for Brazil (INTERBIO) and the United Kingdom (Liverpool), in which the observed associations pointed in the opposite direction (Figure 4). The *I*^2^ statistics indicated low to moderate heterogeneity among the cohorts for both the estimated effect of maternal Cu concentration on PTB (*I*^2^ = 32.8%; *P* = 0.042) and on gestational duration (*I*^2^ = 43.9%; *P* = 0.022). Pooled effect-size estimates were an odds ratio [OR]: 1.30 (95% confidence interval [CI]: 1.08, 1.57) for PTB or 1.64 d (95% CI: 0.56, 2.73) shorter gestation per 1 μg/mL increase in Cu concentration. The association between gestational age at sampling adjusted Cu concentration and gestational duration followed a linear relationship (Supplemental Figure 10) within a wide range of Cu concentration (up to ±3 SD). The fraction of PTB cases also showed a monotonic increase from the lowest to the top quartile group of the adjusted Cu concentration (Supplemental Figure 11).

As maternal Cu concentration changed substantially during pregnancy especially prior to 16 wk of gestation, we performed a subgroup analysis to explore the potential differences in the associations of maternal copper concentration with PTB and gestational duration before and after 16 wk gestation. For maternal samples collected before 16 wk, the estimated effects were OR 1.49 (95% CI: 1.17, 1.89) for PTB or 2.22 d (95% CI: 0.80, 3.64) shorter gestation per 1 μg/mL increase in Cu concentration compared to an OR 1.23 (95% CI: 0.87, 1.75) for PTB and 1.38 d (95% CI: 0.02, 2.75) shorter gestation in samples collected at or after 16 wk (Supplemental Figure 12). Although the association appears somewhat stronger in the samples collected before 16 wk, the confidence intervals substantially overlap. Additionally, a meta-analysis did not find gestational age at sampling to be a statistically significant moderator on the associations with PTB (*P* = 0.39) or gestational duration (*P* = 0.40). We conducted leave-one-out analysis to evaluate whether the observed associations were driven by any particular site. The results demonstrate that each individual site did not influence the overall estimates (Supplemental Figure 13). We performed subgroup analysis of the effect-size estimates based on different study designs (case–control sampling compared with population random sampling, Supplemental Table 2); region of study site (Asian, African and others); and different methods of gestational age dating (i.e., LMP compared with ultrasound), and none of these comparisons showed any significant differences.

### Correlation of maternal Cu concentration with APRs and infections in the Malawi cohort

In the Malawi (iLiNS-DYAD) cohort, we examined correlations between maternal Cu concentration and common analytes including APRs (CRP, AGP, and ALB) and infections (HIV and malaria) in 1239 samples collected at enrollment. Participants of the Malawi cohort were enrolled from 4 health facilities that covered mostly 1 continuous area near Lake Malawi [21]. Some of the common analytes (including CRP, AGP) followed log-normal distributions and were, therefore, log-transformed in the statistical analyses. As significant differences in gestational duration, gestational days at sampling, maternal Cu concentration and many analytes were observed among these 4 Malawi subsites (Supplemental Figure 14), we performed statistical analyses stratified by these 4 subsites using similar methods as we did in the meta-analyses across the 18 major study sites.

**FIGURE 5:**
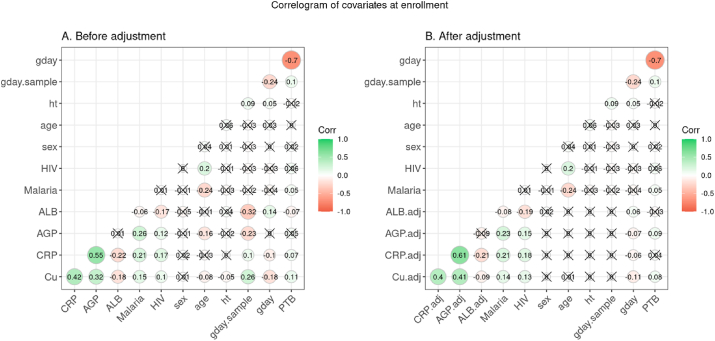
Correlogram of copper (Cu) concentration, acute-phase reactants (APRs), and phenotype measured in the Malawi (iLiNS-DYAD) cohort. Associations between maternal Cu and APRs, infection status were analyzed in 1239 samples from the Malawi cohort.

Similar to the maternal Cu concentration (Figure 3), the concentrations of all the analytes were influenced by gestational age at sampling (Supplemental Figure 15). Therefore, we calculated adjusted values of these variables using the first 2 polynomials of gestational age at sampling and tested their pairwise correlations and their associations with gestational duration and PTB. Strong pairwise correlations were observed among maternal Cu and almost all the APRs, malaria, and HIV infections (Figure 5). These measurements were also significantly correlated with gestational duration or PTB risk. Among these, higher maternal Cu, CRP, and AGP were associated with a higher risk of PTB and shorter gestational duration, and higher ALB was correlated with reduced risk of PTB and longer gestational duration. The infection rates of HIV and malaria were also positively and negatively correlated with maternal age at pregnancy respectively.

The magnitudes of some of the pairwise correlations changed after adjustment for gestational age at sampling (Figure 5). Particularly, the correlations of AGP with PTB or gestational duration were only significant after adjustment, and the correlations of ALB with PTB or gestational duration were less significant after adjustment. After adjustment for gestational age at sampling, the magnitude of the observed association between Cu and AGP increased by 27% and the magnitude of the association of Cu with ALB reduced by 50% (Supplemental Figure 16). Because Cu and the APRs were correlated with each other and all of them were correlated with PTB or gestational duration at different magnitudes (Supplemental Figure 17), we examined whether the observed associations of Cu with PTB and gestational duration were modulated by APRs or HIV and malaria infections. The estimated associations of Cu with PTB and gestational duration were attenuated after the inclusion of other analytes or infections as a covariate, and this was most apparent when adjusting for CRP or AGP (Supplemental Figure 17A). If including all the APRs and infections as covariates (ALL), the effect sizes of Cu reduced substantially; however, the residual association between Cu and gestational duration was still marginally significant (Supplemental Figure 17B).

## Discussion

In this meta-analysis of diverse cohorts from multiple low- and middle-income Asian and sub-Saharan African countries as well as in high-income countries (United Kingdom and United States), we measured maternal prenatal circulating Cu concentrations and tested their association with risk of PTB and gestational duration. Our results demonstrate that maternal Cu concentrations were normally distributed and there was substantial variation among different sites. The lowest Cu concentration was observed in Bangladesh (AMANHI) samples, which was partially due to the early gestational age of sampling at this site (<20 wk). However, even after adjusting for gestational age at sample collection, important differences remained. Maternal Cu levels even differed among sites in geographic and cultural proximity (e.g., 3 Bangladesh sites), indicating that local dietary and environmental factors influence Cu concentrations. We also observed that Cu concentrations were higher in older women, women with shorter stature, and among women gravid with female fetuses. Maternal Cu concentrations increased substantially during early pregnancy—the mean increased by approximately 50% from week 5 to 16 and then reached a plateau around 2.0 μg/mL.

For the association analysis of maternal Cu concentrations (before the third trimester) with PTB and gestational duration, we found significant positive associations between maternal Cu and PTB risk and negative associations with gestational duration. The overall estimated effect sizes are OR 1.30 for PTB risk and 1.64 d shorter gestation per 1 μg/mL increase in maternal Cu concentration. Although among-site heterogeneity was observed, the estimated effects were generally consistent across sites, except for Brazil (INTERBIO) and United Kingdom (Liverpool), in which the associations point in the opposite directions. Overall, the results of our meta-analysis agree with previous studies, which also showed higher maternal Cu levels are associated with increased risk of PTB [18,30]. In addition, we demonstrated that the association between maternal Cu concentration and gestational duration followed a linear relationship, and the PTB rates increased monotonically in mothers with higher quartiles of Cu.

In the Malawi samples (12% of the total samples), higher maternal Cu, CRP, and AGP were associated with a higher risk of PTB and shorter gestational duration, and higher ALB was correlated with reduced risk of PTB and longer gestational duration. These results indicate that Cu could be used as an indicator to capture the associations between acute-phase proteins (e.g., CRP and AGP) and gestational duration.

### Significance and implications of this study

Our findings contribute to an emerging literature focused on the association of Cu status and pregnancy outcomes, especially risk of PTB and gestational duration [17,18,30,31]. Cu is an essential trace element involved in numerous biological processes, and disorders of Cu metabolism in pregnancy, either deficiencies or excesses, can lead to adverse pregnancy outcomes such as preeclampsia and PTB [15]. However, the effect of Cu status on risk of PTB is not well understood, with some suggesting increased PTB and others reporting contradicting results [19,20]. A recent case–control study of pregnant women from Malawi showed that higher maternal Cu at delivery was associated with increased risk of PTB [30]. Hao et al. [18] collected plasma and serum at the first antenatal visit between weeks 4 and 22 of gestation and found that the overall median maternal serum Cu concentrations were significantly higher for preterm births than for term births in the Chinese population. However, there are also reports on gestational length that found contradicting results. Evidence supporting the potential involvement of Cu in PTB risk includes a study of the Maan’Shaan Birth cohort in China, which showed that relatively low umbilical cord Cu levels were associated with higher risk of PTB and early-term birth [19]. These discrepancies may be due to differences in population, study design, maternal or fetal origin of samples, or timing of sampling in pregnancy. Most previous studies have been either case–control studies or mostly focused on a single geographic region and are generally based on small sample sizes.

The present study is the most extensive investigation of the association between early and mid-pregnancy Cu concentration and gestational duration and PTB in global populations, including cohorts from low-income Asian and sub-Saharan African countries with a very high baseline PTB risk. Overall, our results support a consistent association between maternal Cu concentration and PTB and gestational duration. It is possible that Cu may play some functional roles in inflammation and antioxidant mechanisms, the pathways that are implicated in the mechanisms of PTB. Without ceruloplasmin measurements at the same gestational age as Cu measurements, we cannot rule out the confounding effects of the inflammation leading to upregulation of ceruloplasmin, which in turn drives up Cu. We cannot draw causal inference from this observational study, and the observed associations may potentially suggest that Cu is merely a bystander to inflammatory processes. However, even after adjusting for various other inflammatory markers, our results still show that Cu significantly associated with gestational duration suggesting maternal Cu during pregnancy could be a marker of the integrated inflammatory pathways implicated in preterm birth.

The mechanisms underlying the association between higher serum or plasma Cu and PTB are still not fully understood. We propose that maternal circulating Cu concentrations at early or mid-gestation reflect heightened inflammation in those pregnancies destined for spontaneous preterm delivery. The hierarchy of biological activities of Cu calls for biomarkers informative at different levels of Cu exposure assessing Cu intake, placental or tissue Cu, Cu excretion, and Cu biological function. Plasma or serum Cu concentration provides valuable information about the Cu status over a wide range of Cu intake; however, there is need for additional information regarding Cu, particularly for assessing the Cu status and its specific mechanistic role in those at high risk for PTB. Epidemiological reports and research examining the effects of Cu along with specific markers of inflammation and oxidation such as ceruloplasmin, which is Cu-dependent protein, collected at the same gestational age using a standardized protocol are required.

### Strength and limitations

Samples and phenotypic data were retrieved from existing biorepositories collected several years ago in different studies. Although we harmonized and analyzed a set of key variables known to be associated with PTB and gestational duration, we were unable to include some important environmental and socioeconomic factors in the analysis due to missing or incomplete data. We excluded stillbirth due to missing data on cause-of-death, underreporting, and lack of comparability in reporting stillbirths, especially in low- and middle-income countries regarding the birth weight and gestational age criteria. Also, our study only focused on overall PTB, and we have not separated iatrogenic from spontaneous preterm birth as the data are missing from several cohorts. This is a very significant confounder that was not controlled for and likely had an impact on the statistical significance and strength of the association found across the cohorts. It will be key to include these variables across cohorts in future studies.

There were differences in how gestational age was determined and distributed across cohorts. Some cohorts determined the duration by ultrasound fetal biometry (parameters varied across cohorts) whereas others used LMP (or both). This different dating methodology between studies may have introduced some noise into the analysis. PTB rates reported in some low- and middle-income cohort studies appear to be low, and this might be due to underreporting and geographic location of the recruitment site. Also, some cohorts were enriched for PTB samples, and the distribution of gestational duration did not follow a normal distribution. Although regression analysis is generally robust regardless of meeting the normality assumption, and we utilized IPW to adjust for the case/control sampling in regression analysis of gestational duration, this difference in study design and data collection may have introduced some bias in these analyses.

Also, of note with respect to the study limitations is that there was large variation in gestational age when the plasma/serum samples were collected. Given the gestational age at sample collection significantly correlated with the Cu concentration, we accounted for this variance by including only women with samples collected during the first or second trimesters and adjusted for gestational age at sampling in the association analyses. Despite these methodological adaptations, it is possible that we may not have entirely accounted for the influence of gestational age at sample collection. More standardization with respect to the timing of sample collection and storage times may simplify these types of analyses in future studies.

Finally, although we tested several acute-phase proteins (e.g., CRP, AGP, ALB) in Malawi samples, these data were not available in all the study cohorts. Furthermore, we did not measure ceruloplasmin in this current study, which is presumably the most important acute-phase protein that influences the Cu concentration in blood. Future studies of maternal ceruloplasmin and Cu concentration will be essential to elucidate their role in pregnancy.

## Conclusions

Across 18 international birth cohorts with diverse ethnic backgrounds and geographic distribution, there were significant associations between maternal Cu concentration and PTB and gestational duration. These associations were consistent across most study sites, and the association was monotonic and linear across the full range of Cu concentrations. Maternal Cu was strongly correlated with CRP, AGP, and HIV and malaria infections measured in the Malawi samples. Adjustments for these APRs and infections attenuated the observed associations of maternal Cu with PTB and gestational duration, but the associations persist, suggesting that maternal Cu concentration is an indicator of diverse factors that reflect the acute-phase reaction and inflammation that ultimately impact the duration of pregnancy.

## Acknowledgments

We are grateful to all participating families and study personnel from 18 international pregnancy cohorts who took part in this study. Payment for access to data and article-processing charges for this publication was covered by The Bill & Melinda Gates Foundation (Grant no: OPP1175128, OPP1152451). The authors would like to acknowledge the March of Dimes Prematurity Research Center Ohio Collaborative for their support to the original GWAS study that identified the *EEFSEC* gene and led to this study. The Aga Khan University would like to acknowledge Dr Yoshida Sachiyo and Dr Alexander Manu from the World Health Organization, community health workers, pregnant women, and their families. The NEST study acknowledges the support from National Institute of Environmental Health Sciences, the US Environmental Protection Agency, the National Institute of Diabetes and Digestive and Kidney Diseases, and the Duke Cancer Institute. Th CPPOP study acknowledges support from the UCSF California Preterm Birth Initiative. The iLiNS-DYAD-M trial acknowledges support by a grant to the University of California, Davis from The Bill & Melinda Gates Foundation (OPP49817) and a grant to the University of California, Davis from the Office of Health, Infectious Diseases, and Nutrition, Bureau for Global Health, US Agency for International Development (USAID) through the Food and Nutrition Technical Assistance III Project (FANTA). GARBH-Ini cohort acknowledges the support by IUAC for extending Q-ICPMS under National Geochronology Facility funded by Ministry of Earth Sciences (MoES/P.O. (Seismic)8(09)-Geochron/2012). MDIG, AMANHI, GAPPS, and INTERBIO cohorts acknowledge the support by The Bill & Melinda Gates Foundation.

**#**INTERBIO-21st Study Consortium

Jose Villar^1,2^, Rose McGready^3,4^, Fernando C Barros^5^, Cesar G. Victora^6^, Shama Munim^7^, Aris T. Papageorgh^1,2^, Roseline Ochieng^8^, Rachel Craik^1^, Hellen C Barososio^9^, James A. Berkley^10^^,14^, Maria Carvalho^8^, Leila Cheikh Ismail^11^, Ann Lambert^1,2^, Shane A. Norris^12^, Chrystelle OO Tshivuila-Matela^1,12^, Francois Nosten^3,4^, Ricardo Uauy^13^, Zulfiqar A. Bhutta^4^, Stephen Kennedy^1^

^1^Nuffield Department of Women’s & Reproductive Health, University of Oxford, Oxford, United Kingdom; ^2^Oxford Maternal & Perinatal Health Institute, Green Templeton College, University of Oxford, Oxford, United Kingdom; ^3^Shoklo Malaria Research Unit, Mahidol-Oxford Tropical Medicine Research Unit, Faculty of Tropical Medicine, Mahidol University, Mae Sot, Thailand; ^4^Centre for Tropical Medicine and Global Health, Nuffield Department of Medicine, Headington, Oxford, United Kingdom; ^5^Programa de Pós-Graduação em Saúde e Comportamento, Universidade Católica de Pelotas, Pelotas, Brazil; ^6^Programa de Pós-Graduação em Epidemiologia, Universidade Federal de Pelotas, Pelotas, Brazil; ^7^Department of Obstetrics and Gynaecology, Division of Women and Child Health, Aga Khan University, Karachi, Pakistan; ^8^Faculty of Health Sciences, Aga Khan University, Nairobi, Kenya; ^9^KEMRI-Centre for Global Health Research, Liverpool School of Tropical Medicine;^10^KEMRI/Wellcome Trust Research Programme, Kenya; ^11^Clinical Nutrition and Dietetics Department, University of Sharjah, Sharjah, United Arab Emirates; ^12^SAMRC Developmental Pathways For Health Research Unit, Department of Paediatrics & Child Health, University of the Witwatersrand, Johannesburg, South Africa; ^13^Department of Nutrition and Public Health Interventions Research, London School of Hygiene and Tropical Medicine;

### Author contributions

The authors’ responsibilities were as follows—LJM: conceptualized and acquired the funding support for the study; NM, EB, JAL, GFC, JC, GZ, LJM: designed the study; EB, JAL, NM: conducted the ICP-MS analysis of the samples; HX, EB, NM, GZ: compiled the data sets; HX, GZ: developed the analytical pipeline and performed the statistical analysis; JC: coordinated all study related operations; NM, HX, GZ: prepared the first draft of the manuscript; all co-authors: contributed essential intellectual input, revisions of the manuscript, discussed the results, and contributed to the revisions of the final manuscript; NM, GZ: had full access to the data and final responsibility for the decision to submit for publication; and all authors: read and approved the final manuscript.

### Conflict of interest

The authors report no conflicts of interest.

### Funding

This work was supported by the Bill & Melinda Gates Foundation. The funders did not have any role in study design, data analysis, data interpretation, writing of the report, or submission for publication. The findings and conclusions of this article are solely the responsibility of the authors.

Bill & Melinda Gates Foundation (OPP1175128, OPP1152451)

Funders: Cohort Investigations

California Prediction of Poor Outcomes of Pregnancy (CPPOP): UCSF California Preterm Birth InitiativeNEST: National Institute of Environmental Health Sciences (R21ES014947, R01ES016772, P30ES025128, and P01ES022831), the US Environmental Protection Agency (RD-83543701), the National Institute of Diabetes and Digestive and Kidney Diseases (R01DK085173), and the Duke Cancer Institute. iLiNS-DYAD-M trial is funded by a grant to the University of California, Davis from the Bill & Melinda Gates Foundation [OPP49817]. A grant to the University of California, Davis from the Office of Health, Infectious Diseases, and Nutrition, Bureau for Global Health, U.S. Agency for International Development (USAID) under terms of Cooperative Agreement No. AID-OAA-A-12-00005 through the Food and Nutrition Technical Assistance III Project (FANTA), managed by FHI 360.

The Global Alliance to Prevention of Prematurity and Stillbirth (GAPPS) Biorepository Program funded by the Preventing Preterm Birth Initiative grant from the Bill & Melinda Gates Foundation [OPP1033514].

MDIG trial funded by The Bill & Melinda Gates Foundation [OPP1066764]

GARBH – Ini cohort funded by Department of Biotechnology (DBT), Government of India (BT/PR9983/MED/97/194/2013); and Bill & Melinda Gates Foundation (BMGF) (OPP1179761) through Grand Challenges India - Biotechnology Industry Research Assistance Council (GCI -BIRAC) Platform

### Data availability

All deidentified participant data, the statistical code, and technical processes are available from the corresponding author on reasonable request.
